# SERS-Based Immunochromatographic Assay for Sensitive Detection of *Escherichia coli* O157:H7 Using a Novel WS_2_-Au^DTNB^ Nanotag

**DOI:** 10.3390/s25082457

**Published:** 2025-04-14

**Authors:** Deying Wang, Yan Chen, Qi Zhang, Junfei Chen, Changhao Li, Yunjing Luo, Yong Jin, Xiaohua Qi

**Affiliations:** 1 College of Chemistry and Life Sciences, Beijing University of Technology, No. 100 Pingleyuan, Beijing 100124, China; wdyyatb21@163.com (D.W.); llch574751301@126.com (C.L.); 2 Chinese Academy of Inspection and Quarantine, No. A3, Gaobeidian Road, Beijing 100123, China; chenyan6820101@163.com (Y.C.); zhangqi0105@caiq.org.cn (Q.Z.); chenjf@caiq.org.cn (J.C.)

**Keywords:** surface-enhanced Raman spectroscopy, *Escherichia coli* O157:H7, WS_2_-Au, immunochromatographic strip, rapid detection

## Abstract

**Highlights:**

**What are the main findings?**

- Developed a novel WS_2_-Au nanocomposite coupled with 5,5′-dithiobis-(2-nitrobenzoic acid) (DTNB) and antibodies as sensitive surface-enhanced Raman scattering (SERS) nanotags for *E. coli* O157:H7 detection.
- Demonstrated a broad linear detection range (8 × 10^2^–8 × 10^7^ CFU/mL), a low detection limit (175 CFU/mL), high specificity and good accuracy in real food samples (milk, pork).

**What are the implications of the main findings?**

- A rapid assay for qualitative and quantitative detection of *E. coli* O157:H7, overcoming limitations of traditional ICA methods.
- A cost-effective, user-friendly surface-enhanced Raman scattering (SERS)-based immunochromatographic assay (SERS-ICA) platform with great potential for point-of-care testing and detection of other foodborne pathogens.

**Abstract:**

*E. coli* O157:H7 contamination in food and the environment poses a serious threat to human health. Rapid and sensitive identification of foodborne pathogens remains challenging. Here, we prepared tungsten disulfide (WS_2_)–Au nanocomposites coupled with the Raman signal molecule 5,5′-dithio-bis-(2-nitrobenzoic acid) (DTNB) and antibodies to replace the conventional colloidal gold nanoparticles and applied SERS-active nanotags in the SERS-ICA method for highly sensitive detection of *E. coli* O157:H7. The large surface area and numerous effective SERS hotspots of WS_2_-Au nanotags provide superior SERS signals. Under optimized conditions, this ICA achieves the quantitative detection of *E. coli* O157:H7 in a broad linear range of 8 × 10^2^–8 × 10^7^ CFU/mL and at a low detection limit of 175 CFU/mL. In addition, the test strip indicates high specificity for *E. coli* O157:H7 identification, favorable reproducibility, and shows good accuracy in the detection of actual food samples, such as milk and pork. The proposed assay can be used for rapid qualitative and quantitative detection of *E. coli* O157:H7 and has great potential for field application.

## 1. Introduction

*Escherichia coli* O157:H7 (*E. coli* O157:H7), a significant Shiga toxin-producing *E. coli* serotype, is frequently associated with outbreaks. It is prone to contaminating everyday foodstuffs and is transmitted among humans via the fecal–oral transmission route [1,2]. Humans become infected through direct contact with animals harboring the pathogen, their feces, contaminated soil or water, or by consuming undercooked meat products, as well as dairy products, vegetables, and fruits that have been tainted. *E. coli* O157:H7 infections are highly pathogenic and lethal, capable of causing diarrhea and complications such as hemolytic uremic syndrome, severe hemorrhagic diarrhea, and thrombotic thrombocytopenic purpura, posing severe challenges to food hygiene and human health [3,4]. The infective dose of *E. coli* O157:H7 is very low, necessitating strict control in food products [5]. The gold-standard methods for identifying *E. coli* O157:H7 encompass culture isolation, selective enrichment, biochemical identification, and serological typing, which typically require 2–3 days for detection [6]. Modern bacterial detection methods, including polymerase chain reaction (PCR) [7], DNA sequencing [8], and enzyme-linked immunosorbent assay (ELISA) [9], can deliver accurate and sensitive results. However, these methods often involve complex sample preparation steps and rely on sophisticated laboratory equipment, specialized laboratory conditions, and skilled personnel, limiting their suitability for rapid, point-of-care testing [10,11]. Therefore, the development of simple, rapid, and sensitive detection methods for *E. coli* O157:H7 in food samples holds significant scientific and practical importance.

Immunochromatographic assay (ICA) is favored for notable advantages, such as its low cost, ease of operation, rapid detection, providing visible results to the naked eye, and the capability for on-site testing [12,13,14,15]. However, traditional ICA typically utilizes gold nanoparticles as colorimetric labels and relies on visual interpretation for result determination, which is limited by insufficient sensitivity and poor quantitative detection capabilities. To address these limitations, researchers have introduced surface-enhanced Raman scattering (SERS) label-based technology into ICA systems. This integration provides powerful and stable SERS signals, thereby significantly enhancing the accuracy, sensitivity, and quantitative capabilities of the assay. SERS-ICA offers several benefits, including a lower detection limit and the ability to perform quantitative and multiplex detection of various analytes [16,17,18]. In conventional SERS immunochromatography, SERS nanotags modified with specific antibodies bind to the target analytes through antigen–antibody-specific reactions. Highly sensitive quantitative detection of target analytes is achieved by analyzing the characteristic peak intensity of the Raman reporter molecules on the detection line. SERS nanotags primarily consist of three parts: SERS enhancement substrates, Raman reporter molecules, and biomolecular recognition molecules [19,20]. The analytical performance of the SERS-ICA method greatly depends on the SERS enhancement substrates used. The key to developing new SERS nanotags lies in creating stable SERS hotspots to enhance the SERS signals and improve detection sensitivity.

In SERS-ICA, traditional spherical nanomaterials have the disadvantages of easy agglomeration, a low loading capacity, and poor fluidity. In recent years, transition metal dichalcogenides (TMDCs), such as MoS_2_, WS_2_, and MoSe_2_, have been applied in the field of electrochemical, fluorescent, and colorimetric biosensors [21,22,23]. As a typical semiconducting TMDC, WS_2_ possesses many specific characteristics, including a layered structure, a large specific surface area, excellent electronic and optoelectronic properties, and a large direct bandgap, which holds great potential for SERS applications [24,25,26]. The SERS signal of TMDCs is principally attributed to charge transfer processes between the SERS materials and the adsorbed molecules, as well as interfacial dipole–dipole interactions. The enhancement of the SERS signal is dominated by chemical mechanisms [27]. Due to the resonance of near-field and far-field optical properties of TMDCs, the Raman spectral intensity can be enhanced by a factor of 10^2^ through chemical enhancement. Heterostructures and TMDC–precious metal nanocomposites fully co-ordinate the electromagnetic and chemical enhancement mechanisms on the surface of noble metal nanostructures, making them highly promising for developing SERS substrates. Some TMDC–metal heterostructures, such as Ag@WS_2_, AuNPs/MoS_2_/graphene, and AuNPs@DA@MoS_2_, exhibit significant SERS enhancement effects [28,29,30,31].

In this study, we proposed WS_2_-Au SERS labels and incorporated them into SERS-ICA for the ultrasensitive and quantitative determination of the common foodborne bacteria *E. coli* O157:H7 in food samples. In the WS_2_-Au nanocomposites, WS_2_ nanosheets were utilized as a support material for AuNPs, providing abundant anchoring sites and thus efficiently capturing the target pathogens. The strong coupling between WS_2_ and Au nanostructures through surface plasmon excitation could be beneficial to further enhance the effectiveness of SERS [32,33,34]. Through conditional optimization, the established assay enabled the sensitive identification of *E. coli* O157:H7 using a single SERS-ICA strip within 20 min. The limit of detection (LOD) for *E. coli* O157:H7 was 175 CFU/mL. The proposed WS_2_-Au-based SERS-ICA demonstrated high reproducibility and reliability in real food samples, such as milk and pork. Hence, the WS_2_-Au-based SERS-ICA holds significant promise for the rapid and sensitive detection of bacteria in the POCT field.

## 2. Materials and Methods

### 2.1. Reagents and Equipment

Nitrocellulose (NC) membranes (Sartorius Stedim UniSart CN140, Sartorius, Göttingen, Germany), sample pads (Millipore GFCP20300, Millipore, Billerica, MA, USA), absorbent pads (Millipore CFSP223000, Millipore, Billerica, MA, USA), and polyvinyl chloride (PVC) base plates were purchased from Shanghai Jinbiao Biotechnology Co., Ltd., Shanghai, China. Tetrachloroaurate (III) trihydrate (HAuCl_4_·3H_2_O, 98%), trisodium citrate (Na_3_C_6_H_5_O_7_·2H_2_O, 99.8%), sodium dihydrogen phosphate (NaH_2_PO_4_, 99%), potassium dihydrogen phosphate (KH_2_PO_4_, 99%), potassium carbonate (K_2_CO_3_, 99.0%), sodium chloride (NaCl, 99%), potassium chloride (KCl, 99%), borax (Na_2_B_4_O_7_·10H_2_O, 99.5%), boric acid (H_3_BO_3_, AR), bovine serum albumin (BSA, 98%), and Proclin (Proclin^TM^300) were bought from Sigma Corporation, St. Louis, MO, USA. Monolayer WS_2_ nanosheets were bought from Beike 2D materials Co., Ltd., Beijing, China. *E. coli* O157:H7 monoclonal antibody (mAb) and goat anti-mouse IgG antibody were provided by our laboratory. 5,5′-dithiobis-(2-nitrobenzoic acid) (DTNB, 99%) and Tween-20 were obtained from Aladdin Reagent Co., Ltd., Shanghai, China. All other chemicals were of analytical grade. Food samples, including milk and pork, were purchased from the local supermarket.

A UV-Vis spectrophotometer (U-3310, Hitachi Ltd., Tokyo, Japan) was used to obtain UV–visible absorption spectra. A transmission electron microscope (JEM-2100F, 200 kV; JEOL Ltd., Tokyo, Japan) was used to acquire TEM images. Elemental mapping of the substrates was performed using an energy-dispersive X-ray analyzer (7593-H; Horiba Ltd., Northampton, UK). A three-dimensional spraying instrument (XYZ 3050; Biodot Ltd., Irvine, CA, USA) was used to spray the antibody onto the NC membrane, and a programmable cutting machine (CM 4000, Biodot Ltd., Irvine, CA, USA) was used to cut the test strips. The SERS intensity was measured by a laser confocal microscope Raman spectrometer (InVia Reflex; Renishaw, UK), recording laser excitation wavelengths under 785 nm, with a diffraction grating of 1200 grooves/mm, an exposure time of 4 s, a laser output power of 1 mW, and 50× objective lens focusing.

### 2.2. Synthesis of WS_2_-Au^DTNB^ Nanotags

**Figure 1:**
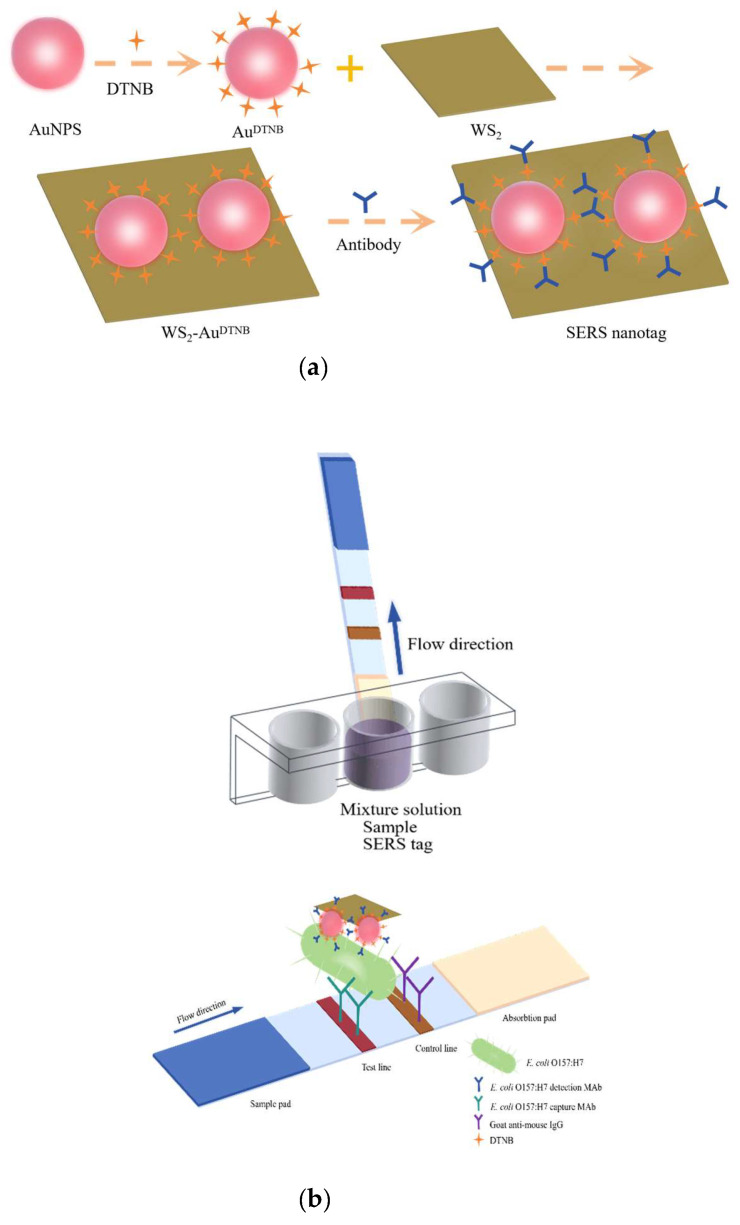
Schematic illustration of the fabrication procedure of SERS-ICA. (**a**) Preparation and antibody modification of WS_2_-Au^DTNB^ SERS nanotags. (**b**) ICA strip procedure for *E. coli* O157:H7 detection.

The preparation process of the SERS nanotags is illustrated in Figure 1a. AuNPs were synthesized through a modified trisodium citrate reduction method [35]. Briefly, 1 mL of 1% (*w*/*v*) HAuCl_4_·3H_2_O solution was added to 99 mL of deionized water and stirred in a microwave synthesizer. When the temperature rose to 98 °C, it was maintained stably for 2 min, after which 1.2 mL of 1% (*w*/*v*) trisodium citrate solution was rapidly added. The solution was then boiled for an additional 5 min. Once the solution turned wine-red, it was removed from the heat, cooled to room temperature, and then stored in a 4 °C refrigerator. Next, the Raman reporter molecule DTNB (20 μL, 10 mM) was added to 10 mL of the prepared AuNP solution and stirred continuously for 2 h at room temperature. Excess DTNB was removed by centrifugation (12,000 rpm, 10 min), and the precipitate was resuspended in 2 mL of ultrapure water. Subsequently, 2 mL of Au^DTNB^ solution was mixed with 2 mL WS_2_ nanosheets (1 mg/mL), and the mixture was ultrasonicated for 30 min, followed by overnight stirring. The formed WS_2_-Au nanocomposites were centrifuged (12,000 rpm, 10 min) and resuspended in 2 mL of ultrapure water.

### 2.3. Preparation of Antibody-Modified WS_2_-Au^DTNB^ Nanotags

A quantity of 10 µL of 0.05 M K_2_CO_3_ was added to the 1 mL prepared WS_2_-Au^DTNB^ solution to adjust the pH, then incubated with the 0.2 mg/mL *E. coli* O157:H7 detection monoclonal antibody (mAb) for 30 min. The mixture was added with 100 μL of 10 % BSA (*w*/*v*) and vortexed for 30 min to block the unreacted carboxyl sites on the surface of the SERS nanotags. Finally, antibody-modified WS_2_-Au^DTNB^ SERS nanotags were collected by centrifugation (12,000 rpm, 10 min) and resuspended in 100 μL of antibody preservation solution containing 0.1 M boric acid-buffered solution, 10% (*w*/*v*) BSA, and proclin.

### 2.4. SERS-ICA Test Strip Assembly

*E. coli* O157:H7 capture antibody and goat anti-mouse IgG antibody were sprayed onto the NC membrane to form the detection line (T-line) and the control line (C-line), respectively. The sprayed NC membrane was then dried overnight in an oven at 37 °C. The sample pad was treated with 0.01 M Tris solution (containing 0.5% Tween-20 (*w*/*v*), pH 8.0) and subsequently dried. To assemble the ICA strips, the treated NC membrane was affixed to the center of the PVC backing plate, with the sample pad and absorbent pad placed on either side of the NC membrane. The assembled card was cut into 4 mm wide strips using the CM400 programmable cutting machine and stored in a desiccant-sealed bag.

### 2.5. WS_2_-Au^DTNB^-Based ICA Strips for Analysis of E. coli O157:H7

Standard *E. coli* O157:H7 bacterial strains were provided by the Testing and Evaluation Center’s laboratory at the Chinese Academy of Inspection and Quarantine. Different concentrations of standard bacterial solutions, ranging from 8 × 10^2^ to 8 × 10^7^ CFU/mL, were diluted in blocking solution. Then, 6 μL of immunoprobe (WS_2_-Au^DTNB^) was mixed with 100 μL of *E. coli* O157:H7 standard solution in a 96-well plate, and the strips were inserted for the reaction. As the reaction proceeded, the T-line and C-line began to show a dark-purple color visible to the naked eye, and the entire reaction process was completed within 20 min. The SERS signals arising from the DTNB embedded in the immunoprobe captured by the detection antibody on the T-line could be measured with the confocal microscopic Raman spectrometer.

### 2.6. Spiked Sample Preparation and Detection

Milk and pork were used to evaluate the practical application of this WS_2_-Au based SERS-ICA. *E. coli* O157:H7 standard solutions were spiked into pork and milk to prepare 8 × 10^4^ to 8 × 10^6^ CFU/mL spiked samples. For pork samples, 50 µL of *E. coli* O157:H7 solution was added to 4 g of minced meat, followed by the addition of 4 mL of PBS buffer. The mixture was vortexed for 10 min on a shaker and then centrifuged (10,000 rpm, 10 min) to obtain the supernatant. For milk samples, 10 µL of *E. coli* O157:H7 solution was mixed with 990 µL of milk and thoroughly mixed. These spiked samples were tested using the WS_2_-Au based ICA, adhering to the previously outlined analytical method.

## 3. Results

### 3.1. Characterization of Au, WS_2_-AuNPs, and SERS Nanotags

**Figure 2:**
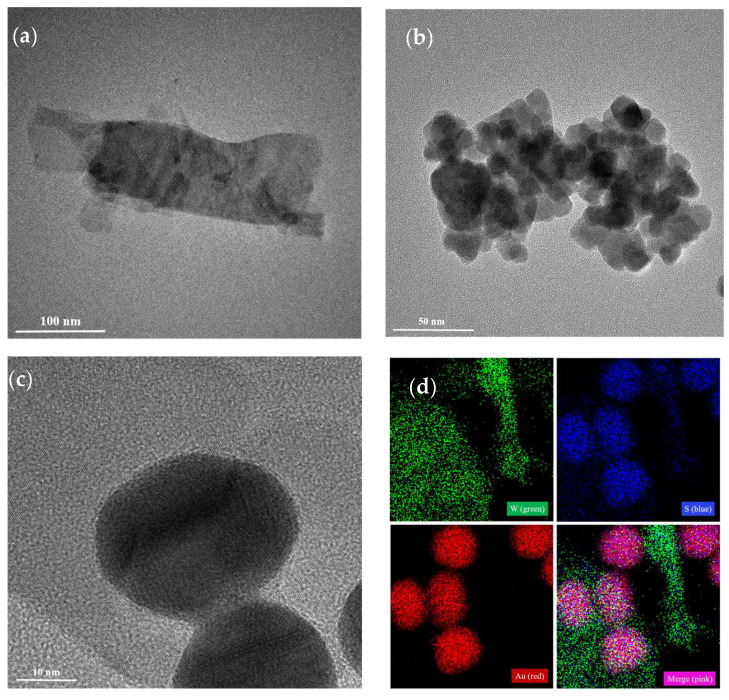
Characterization of the fabricated WS_2_-Au complexes. TEM images of (**a**) WS_2_ and (**b**) WS_2_-Au. (**c**) HRTEM images of AuNPs on the WS_2_. (**d**) EDX elemental (W, S, Au, and merging) mapping images of the WS_2_-Au.

The formation of the WS_2_-Au nanocomposites was based on the Hard and Soft Acids and Bases (HSAB) theory, according to which the soft sulfur surface layer of WS_2_ binds with the soft acid Au nanoparticles [36,37]. The morphologies and structures of the WS_2_-Au nanocomposites were investigated by TEM. The AuNPs possessed a uniform spherical morphology, with an average size of 26.65 nm, as measured from TEM images using ImageJ 2025 software (Figure S1). As shown in Figure 2a, the monolayer WS_2_ nanosheet exhibited a thin layer of a wrinkled structure with a large surface area and good dispersion. In the case of the WS_2_-Au nanocomposites (Figure 2b,c), the Au nanoparticles were integrated onto the surface of the WS_2_ nanosheets. Figure 2d exhibits the element mapping results of the WS_2_-Au nanocomposites; the distribution of W (green), S (blue), and Au (red) on the nanosheet and their merging were demonstrated clearly. The UV-Vis absorbance spectra were measured to characterize the localized surface plasmon resonance (LSPR) properties of the AuNPs and WS_2_-Au^DTNB^, as shown in Figure S2. The ultraviolet absorption at 529 nm corresponds to the strong LSPR band of the Au nanoparticles. The absorption spectrum of WS_2_ shows broad absorption in the visible region, primarily due to exciton transitions in WS_2_. For WS_2_-Au, the absorption peak displayed a modest red shift and an expansion of the absorption peak [38]. The above results demonstrate the successful formation of the WS_2_-Au nanocomposites. Based on the Tauc plot of WS_2_ shown in Figure S3, the band gap of the WS_2_ nanosheet was estimated to be 2.09 eV.

The SERS activities of the Au and WS_2_-Au were investigated by DTNB. As revealed in Figure S4, many more fold-enhanced Raman signals of 10^−6^ M DTNB were detected on the WS_2_-Au SERS platform than those of Au nanoparticles. This enhancement was attributed to the synergistic effect of the chemical enhancement provided by the WS_2_ nanosheets and the electromagnetic enhancement between Au and WS_2_.

### 3.2. Construction of WS_2_-Au-Based ICA for Determination of E. coli O157:H7

The principle of the SERS-ICA test strips for detecting *E. coli* O157:H7, based on the double-antibody sandwich method, is schematically illustrated in Figure 1b. The WS_2_-Au^DTNB^ nanotags were mixed and bound with the *E. coli* O157:H7 sample solution in the microwell to form a complex and then dipped into test strips. Under the action of capillary chromatography, the formed nanotag–*E. coli* O157:H7 antigen complex was captured by the immobilized antibody as it flowed to the T-line, while the remaining uncaptured complexes continued to flow to the C-line and were captured by the goat anti-mouse antibody. Ultimately, the targets could be qualitatively observed through color changes in the test zone and quantitatively analyzed by measuring the SERS signal intensity on the T-line.

### 3.3. Optimization of the Assay Conditions

To establish a more sensitive and accurate SERS-ICA analysis method, the pH value of the solution and the amount of detection antibody during the preparation of the WS_2_-Au^DTNB^ nanotags, as well as the coating antibody concentration, were optimized. The effects of different level parameters were assessed by measuring the Raman signal intensity on the T-line.

**Figure 3:**
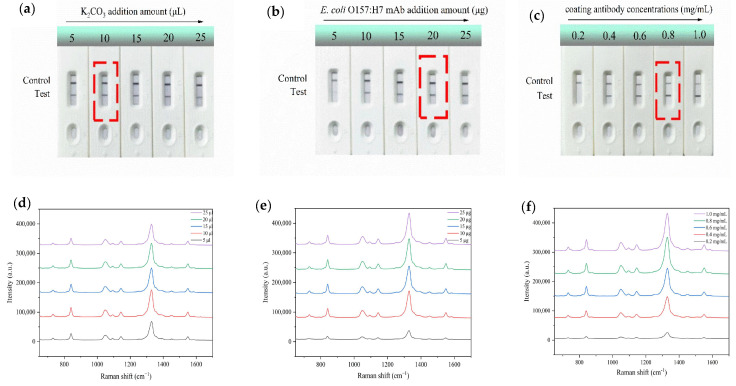
Images of optimized (**a**) pH value, (**b**) *E. coli* O157:H7 mAb, and (**c**) coated antibody concentrations. SERS intensities of different (**d**) K_2_CO_3_ volumes, (**e**) *E. coli* O157:H7 mAb addition amounts, (**f**) and coated antibody concentrations.

The pH value is a key factor influencing SERS nanotag fabrication, which affects the aggregation of nanotags. By adjusting the pH using 0.05 M K_2_CO_3_ solution, the addition amount was varied from 5 to 25 μL. Figure 3a shows that with the addition of K_2_CO_3_ solution, the color development of the T-lines on the test strip gradually intensifies. When the amount of K_2_CO_3_ added reaches 10 μL, the color of the T-lines on the test strip is the deepest. However, if the amount of K_2_CO_3_ solution is further increased, the color of the T-lines on the test strip gradually fades. Excessive K_2_CO_3_ solution destabilizes the electrical double layer of AuNPs, leading to their aggregation and preventing effective migration to the T-line. Additionally, the elevated pH may impair antibody activity, ultimately resulting in weakened color development on the T-line. Combined with the SERS measurement results in Figure 3d, the SERS intensity is highest when the amount of K_2_CO_3_ added is 10 μL. Herein, 10 μL of K_2_CO_3_ was applied for further experiments.

In addition, the amount of 0.2 mg/mL antibody for SERS nanotag preparation was optimized. As indicated in Figure 3b, with increasing amounts of *E. coli* O157:H7 mAb antibody, the T-lines’ color on the strips gradually became darker. The SERS intensity, as measured in Figure 3e, reached a maximum when the antibody amount was 20 μg. Therefore, 20 μg of antibody was chosen as the optimal amount for preparing WS_2_-Au^DTNB^-Ab.

Finally, the concentration of the coating antibody on the NC membrane was optimized. Figure 3c,f illustrate that as the antibody concentration increased from 0.2 mg/mL to 0.8 mg/mL, the color and intensity of the T-line increased rapidly and then showed a slight increase, indicating saturation of the antibody and bacterial immune response. Consequently, the optimal concentration for the coating antibody was identified as 0.8 mg/mL.

### 3.4. Sensitivity of the Assay

In order to evaluate the sensitivity of the SERS-ICA, a series of *E. coli* O157:H7 samples with a range of different concentrations (8 × 10^2^–8 × 10^7^ CFU/mL) were applied to SERS-ICA procedures under the above optimized conditions.

**Figure 4:**
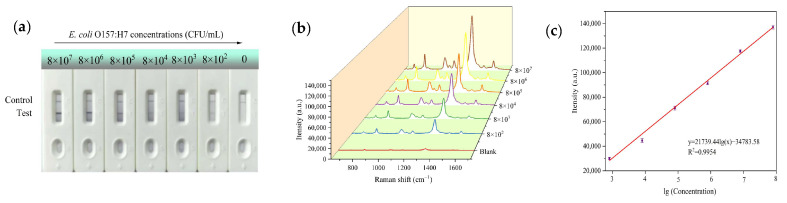
(**a**) Images of SERS-ICA detection of *E. coli* O157:H7 standard bacterial solutions at different concentrations. (**b**) SERS spectra for different concentrations of *E. coli* O157:H7. (**c**) SERS signal intensities at 1329 cm^−1^ increasing linearly with logarithmic concentration of *E. coli* O157:H7.

For qualitative analysis, Figure 4a displays the WS_2_-Au-based SERS-ICA strip for *E. coli* O157:H7 detection at various concentrations. Visual qualitative analyses were performed by three independent observers to identify a visible color signal distinct from blank negative controls (blanks). Under standardized white-light illumination, it was observed that the concentration of *E. coli* O157:H7 decreased and the dark-purple color of the T-line gradually faded due to fewer nanoprobes being captured at the T-line. The visual sensitivity, consistently observed by the naked eye, was estimated to be 8 × 10^2^ CFU/mL. SERS spectra of different *E. coli* O157:H7 concentrations were measured for quantitative detection. As shown in Figure 4b, the SERS signal intensity gradually increased with the *E.coli* O157:H7 concentration. The characteristic peak intensities of DTNB at 1329 cm^−1^ could be employed as a basis for the standard curve of the SERS-based ICA. In Figure 4c, the curve demonstrates a good linear relationship within the concentration range of 8 × 10^2^ CFU/mL to 8 × 10^7^ CFU/mL. The linear equation at 1329 cm^−1^ can be described as y = 21739.44 lg(x) − 34783.58, with a correlation coefficient of R^2^ = 0.9954, which can provide a reliable basis for determination of *E.coli* O157:H7 in quantitative analysis. On the basis of the formula LOD = y_blank_ + 3 × SD_blank_, where y_blank_ and SD_blank_ are the average Raman intensity and standard deviation of the blank control, respectively [39], the LOD was calculated as 175  CFU/mL.

### 3.5. Specificity and Repeatability Evaluations

**Figure 5:**
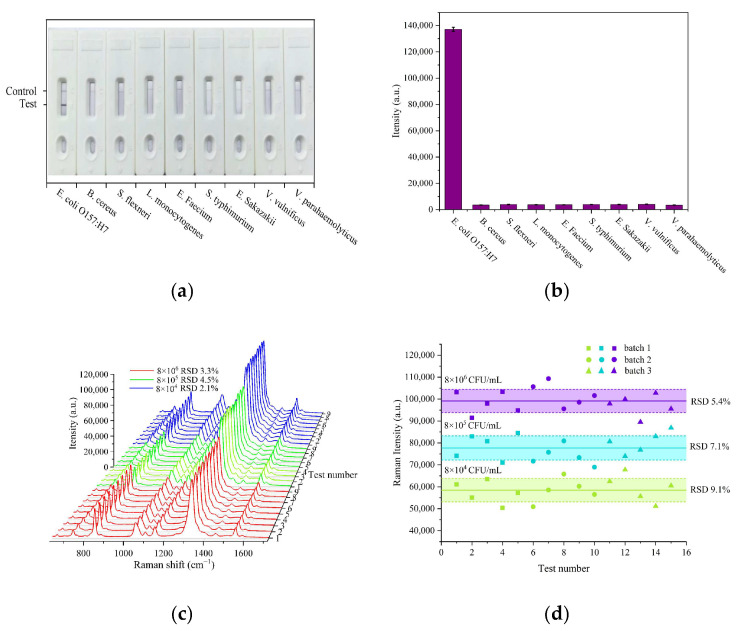
Selectivity and reproducibility of the developed ICA for *E. coli* O157:H7. (**a**) Digital photograph of 9 strips of different bacteria. (**b**) Corresponding Raman intensities at 1329 cm^−1^. (**c**) SERS signals of nine tests of *E. coli* O157:H7. (**d**) SERS intensities at 1329 cm^−1^ measured in three different batches of strips.

The specificity and repeatability of this ICA strip were evaluated using eight other pathogenic bacteria, including *Bacillus cereus*, *Shigella flexneri*, *Listeria monocytogenes*, *Enterococcus faecium*, *Salmonella typhimurium*, *Enterobacter sakazakii*, *Vibrio vulnificus*, and *Vibrio parahaemolyticus*, as interfering strains for SERS-ICA detection. As shown in Figure 5a,b, only the assay for *E. coli* O157:H7 generated a distinct color band and showed strong SERS signals in the corresponding T-lines, while no obvious color band or SERS intensity appeared on the T-lines for other non-target bacteria. This result is related to the specific affinity of the antibodies coated on the NC membranes for *E. coli* O157:H7. Compared to other reported methods, as shown in Table S1, the WS_2_-Au-based SERS-ICA strips significantly improve the detection sensitivity for the target bacteria. To assess the reproducibility, SERS intensities on the T-line of 8 × 10^6^, 8 × 10^5^, and 8 × 10^4^ CFU/mL of *E. coli* O157:H7 were tested at nine different points, with RSDs of 3.3%, 4.5%, and 2.1% (Figure 5c). Batch-to-batch reproducibility was evaluated using strips prepared in three different batches (Figure 5d). The RSDs for these tests were 5.4%, 7.1%, and 9.1% for 8 × 10^6^, 8 × 10^5^, and 8 × 10^4^ CFU/mL of *E. coli* O157:H7, respectively. These results confirmed the good specificity and reproducibility of the proposed assay.

### 3.6. Detection of E. coli O157:H7 in Spiked Milk and Pork Samples

**Figure 6:**
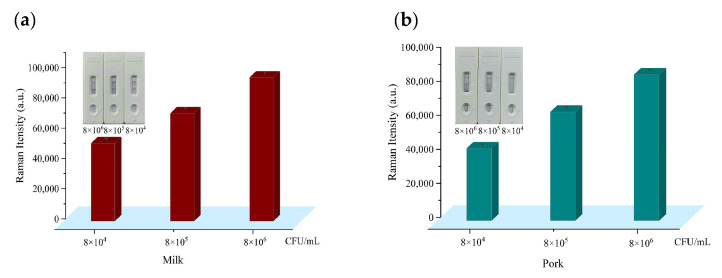
Practicability test of the WS_2_-Au-based ICA for *E. coli* O157:H7 detection in (**a**) milk and (**b**) pork samples.

It is extremely essential to investigate the practicability of the proposed SERS-based ICA for sample analysis. Accordingly, pasteurized milk and pork samples obtained from a supermarket were spiked with *E. coli* O157:H7 standard solution and applied to the proposed assay under the optimized conditions, and the results are shown in Figure 6a,b. The contents of *E. coli* O157:H7 in the spiked samples were quantified based on the standard curve. The recoveries of *E. coli* O157:H7 from the spiked samples measured by SERS-based ICA are presented in Table S2. The average recoveries for *E. coli* O157:H7 pathogens in milk and pork were 95.3–117.6% and 86.8–101.5%, respectively, with RSDs ranging from 2.02% to 9.52%. These results indicate that this novel strategy is a feasible approach to effectively detect *E. coli* O157:H7 in real food samples.

## 4. Conclusions

Due to the high SERS enhancement, good biocompatibility, and high stability of the designed WS_2_-Au^DTNB^ high-performance SERS nanotags, we have successfully developed a low-cost and user-friendly SERS-ICA platform for rapid and sensitive detection of *E. coli* O157:H7. The assay utilizes immune complex formation on the T-line, where the SERS signal intensity at 1329 cm^−1^ (DTNB) correlates with bacterial concentration. Under optimal experimental conditions, this method achieves a detection limit of 175 CFU/mL, with the entire assay completed within 20 min. The SERS-ICA possesses satisfactory sensitivity, specificity, and reproducibility, as well as an acceptable recovery rate (86.8–117.6%), in real food samples. This proposed method proves to have important prospects in point-of-care testing and can be used for the detection of other pathogens by coupling corresponding specific antibodies.

Future research can prioritize enhancing detection sensitivity through rational design of SERS-active substrates, where hierarchical nanostructures and strategic integration of semiconductor materials with metallic nanoparticles can generate high-density electromagnetic hotspots for amplified Raman signals. Concurrently, portable device integration will be advanced by coupling SERS readouts with smartphone-based spectral analyzers, while multiplexed pathogen identification in complex matrices necessitates further exploration. Collectively, these innovations aim to merge laboratory-grade analytical precision with field-operational simplicity, addressing critical gaps in foodborne pathogen monitoring.

## Data Availability

The data presented in this study are available upon request from the corresponding author.

## Supplementary Materials

The following supporting information can be downloaded at https://www.mdpi.com/article/10.3390/s25082457/s1: Figure S1: The Au size distribution obtained by Image J 2025 software; Figure S2: UV-vis absorption spectra of AuNPs, WS_2_, and WS_2_-Au; Figure S3: Tauc plot of WS_2_; Figure S4: SERS spectra of 10^−6^ M DTNB molecules on Au and WS_2_-Au; Table S1: Comparison with other techniques for bacteria detection; Table S2: Recoveries of *E. coli* O157:H7 spiked in milk and pork. References [40,41,42,43,44,45] are cited in the supplementary materials.
