# Supplementary material for: SERS-Based Immunochromatographic Assay for Sensitive Detection of Escherichia coli O157:H7 Using a Novel WS2-AuDTNB Nanotag
Source: Sensors (Basel). 2025 Apr 14;25(8):2457. doi: 10.3390/s25082457 (PMC12031149; doi:10.3390/s25082457)
Supplement: Supplementary file 1 [file sensors-25-02457-s001.zip › sensors-3534159-supplementary.docx]

**Supporting Information**

**SERS-Based Immunochromatographic Assay for Sensitive**

**Detection of *Escherichia coli* O157:H7 Using a Novel**

**WS_2_-Au^DTNB^ Nanotag**

Deying Wang^1,2^ Yong Jin^2^ Qi Zhang^2^ Junfei Chen^2^ Yan Chen^2^ Changhao Li^1,2^ Yunjing Luo^1^* and Xiaohua Qi^2^*

1. College of Chemistry and Life Sciences, Beijing University of Technology, No. 100 Pingleyuan, Beijing 100124, China
2. Chinese Academy of Inspection and Quarantine, No. A3, Gaobeidian Road, Beijing 100123, China

*Corresponding Authors

Yunjing Luo, Email: [luoyj@bjut.edu.cn](mailto:luoyj@bjut.edu.cn)

Yong Jin, Email: jinyongdr@163.com

Xiaohua Qi, Email: [qixh2000@126.com](mailto:qixh2000@126.com)


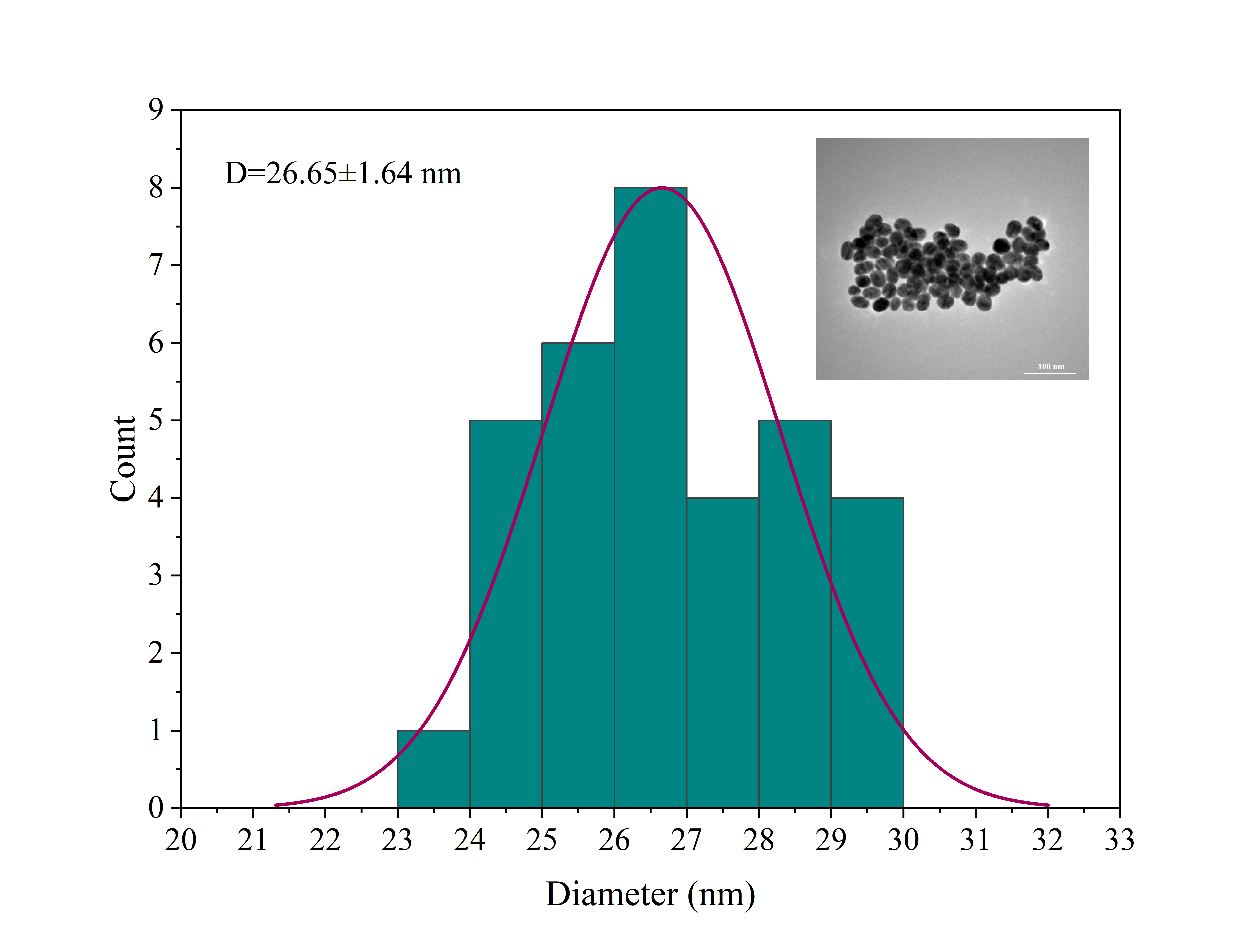


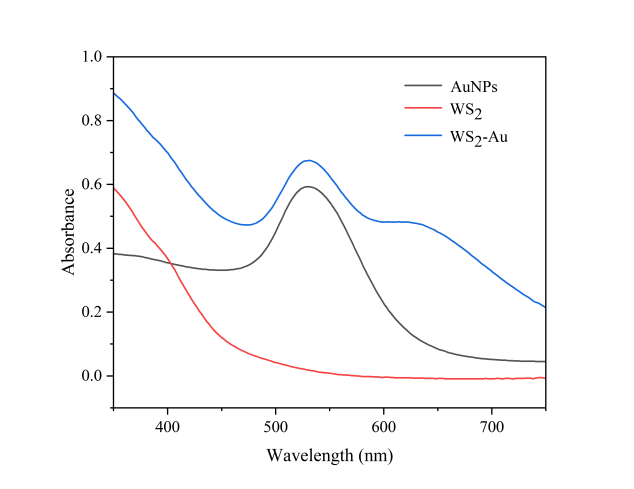
Figure. S1 The Au size distribution obtained by Image J 2025 software

Figure. S2 UV-vis absorption spectra of AuNPs, WS_2_, WS_2_-Au


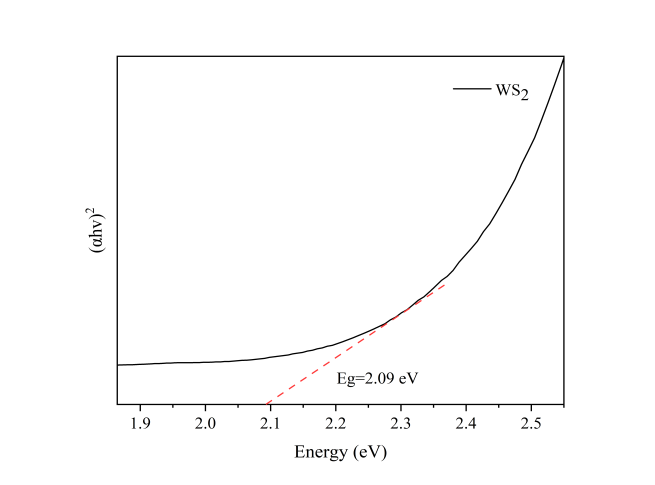


Figure. S3 Tauc plot of WS_2_


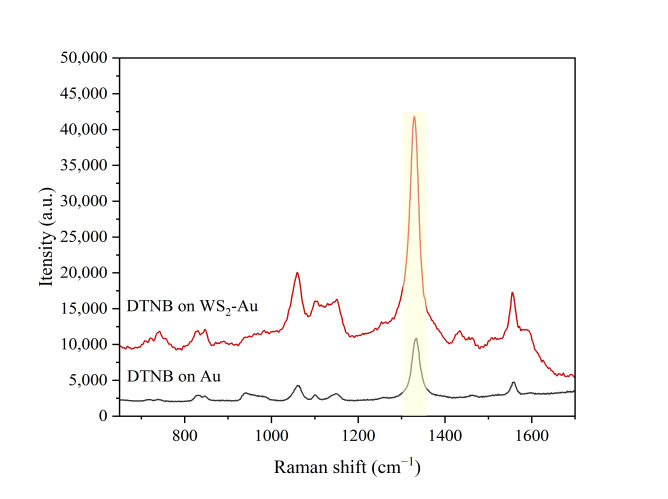


Figure. S4 SERS spectra of 10^−6^ M DTNB molecules on Au, WS_2_-Au

Table S1 Comparison with other techniques for bacteria detection

| **Methods** | **Lables** | **LOD(CFU/mL)** | **Samples** | **Reference** |
| --- | --- | --- | --- | --- |
| Colorimetric aptasensor | G-triplex/hemin DNAzyme | 1.3× 10^3^ | milk | [1] |
| SERS-LFA | Au^MBA^@Ag | 5×10^4^ | milk, chicken breast, beef | [2] |
| Immunoassay | nanobodies | 8.7×10^3^ | ground beef, orange juice, milk | [3] |
| Fluorescent immunoassay | FL-BAPFNPs | 306 | milk, beer, and beef | [4] |
| Electrochemical sensor | anti-microbial peptides (AMPs) | 3.4 | milk | [5] |
| Bacteria-imprinted impedimetric (BIP) sensor | PPy/CuPcTs | 21 | drinking water, orange juice, milk | [6] |
| SERS-ICA | WS_2_-Au^DTNB^ | 175 | milk, pork | This work |

Table S2. Recoveries of *E.coli* O157:H7 spiked in milk and pork

| **Sample** | **Spiked (CFU/mL)** | **Measured**  **(CFU/mL)** | **Recovery (%)** | **RSD (%, n=3)** |
| --- | --- | --- | --- | --- |
| Milk | 8×10^6^ | 9.41×10^6^ | 117.6 | 7.68 |
|  | 8×10^5^ | 7.62×10^5^ | 95.3 | 2.02 |
|  | 8×10^4^ | 9.35×10^4^ | 116.9 | 5.44 |
| Pork | 4×10^6^ | 3.57×10^6^ | 89.3 | 6.13 |
|  | 4×10^5^ | 3.47×10^5^ | 86.8 | 9.52 |
|  | 4×10^4^ | 4.06×10^4^ | 101.5 | 4.78 |

**References**

[1] Pang, L.; Wang, L.; Liang, Y.; Wang, Z.; Zhang, W.; Zhao, Q.; Yang, X.; Jiang, Y. G-triplex/hemin DNAzyme mediated colorimetric aptasensor for Escherichia coli O157:H7 detection based on exonuclease III-assisted amplification and aptamers-functionalized magnetic beads. *Talanta* **2024**, *269*, 125457.

1. Liu, H. B.; Chen, C. Y.; Zhang, C. N.; Du, X. J.; Li, P.; Wang, S. Functionalized Au^MBA^@Ag Nanoparticles as an Optical and SERS Dual Probe in a Lateral Flow Strip for the Quantitative Detection of Escherichia coli O157:H7. *J. Food Sci*. **2019**, *84*, 2916–2924.
2. He, Q.; Pan, J.; Xu, Z.; Hammock, B. D.; Li, D. Development of a nanobody-based immunoassay for the detection of Escherichia coli O157:H7 in food samples. *Food Chem*. **2025**, *473*, 142987.
3. Zhang, G.; Huang, Z.; Hu, L.; Wang, Y.; Deng, S.; Liu, D.; Peng, J.; Lai, W. Molecular Engineering Powered Dual-Readout Point-of-Care Testing for Sensitive Detection of Escherichia coli O157:H7. *ACS Nano*. **2023**, *17*, 23723–23731.
4. Kim, J. E.; Shin, J. H.; Park, J. P. An engineered antimicrobial peptide as an alternative bioreceptor for the detection of pathogenic Escherichia coli O157:H7. *J. Electroanal. Chem*. **2024**, *953*, 118003.
5. Zhu, M.; Liu, J.; Jiang, X.; Zhang, Y.; Zhang, J.; Wu, J. Bacteria-imprinted impedimetric sensor based on doping-induced nanostructured polypyrrole for determination of Escherichia coli. *Mikrochim. Acta*. **2023**, *190*, 431.
